# Compartment syndrome in the first dorsal interosseous compartment: case report

**DOI:** 10.1590/1677-5449.200094

**Published:** 2021-04-02

**Authors:** Clara Wilma Fernandes Rosendo, José Rodolfo Lopes de Paiva Cavalcanti, Raimundo Rosendo de Oliveira

**Affiliations:** 1 Universidade Federal do Rio Grande do Norte – UFRN, Departamento de Ciências da Saúde, Natal, RN, Brasil.; 2 Universidade do Estado do Rio Grande do Norte – UERN, Mossoró, RN, Brasil.; 3 Hospital Regional Tarcísio Maia – HRTM, Departamento de Cirurgia Geral e Vascular, Mossoró, RN, Brasil.

**Keywords:** compartment syndromes, fasciotomy, burns

## Abstract

In areas that are not commonly affected by compartment syndrome because they have a good content/container ratio, diagnosis of the condition can be a challenge, since surgeons will find it difficult to make a diagnosis on the basis of an isolated sign or symptom. As a result, the correct treatment can very often be delayed, causing harm to the patient. In this case, the patient was a 29-year-old woman who was seen for a painful left hand secondary to a large burn injury to the area anterior of the anatomical snuffbox. She had already undergone surgery in her home town 30 days previously, with debridement of skin and subcutaneous tissue, but the pain had not improved. She was on antibiotic therapy (ceftriaxone, 1g every 12 hours) and analgesia, with therapeutic morphine doses every 8 hours.

## INTRODUCTION

References on compartment syndrome cover involvement of muscle and nerve structures that are weakened by motor and sensory dysfunction. This pathological condition is caused by an imbalance between content and container, causing injury to these structures and, if not properly and promptly treated, it results in irreversible sequelae that depend on the degree of compromise.

Vascular surgeons are primarily used to encountering this pathology in the upper and lower limbs. However, it is possible for this syndrome to involve any part of the body. Diagnosis is based on a careful clinical assessment and does not normally require specialized diagnostic methods. The primary objective is to avoid serious complications, such as motor dysfunction.

## CASE DESCRIPTION

This study complies with the recommendations set out in CNE resolution 466/2012. It was duly approved by the Research Ethics Committee (CAAE 31017920.7.0000.5294 and protocol 4.050.507).

The patient was a 29-year-old female who presented at the emergency room complaining of intense pain in the left hand caused by a hot coffee scald. At the time of presentation, she was already taking morphine for symptom relief. On physical examination, she had a large injury at the level anterior of the anatomic snuffbox, with a rough crust covering the entire wound, signs of edema of the hand, and functional weakness of the fingers, in addition to intense pain in response to manipulation.

Four weeks previously, the patient had undergone surgical debridement of the wound in her home town, but the pain had not abated and she was on continuous morphine, without satisfactory results. There were no signs of local infection.

**Figure 1:**
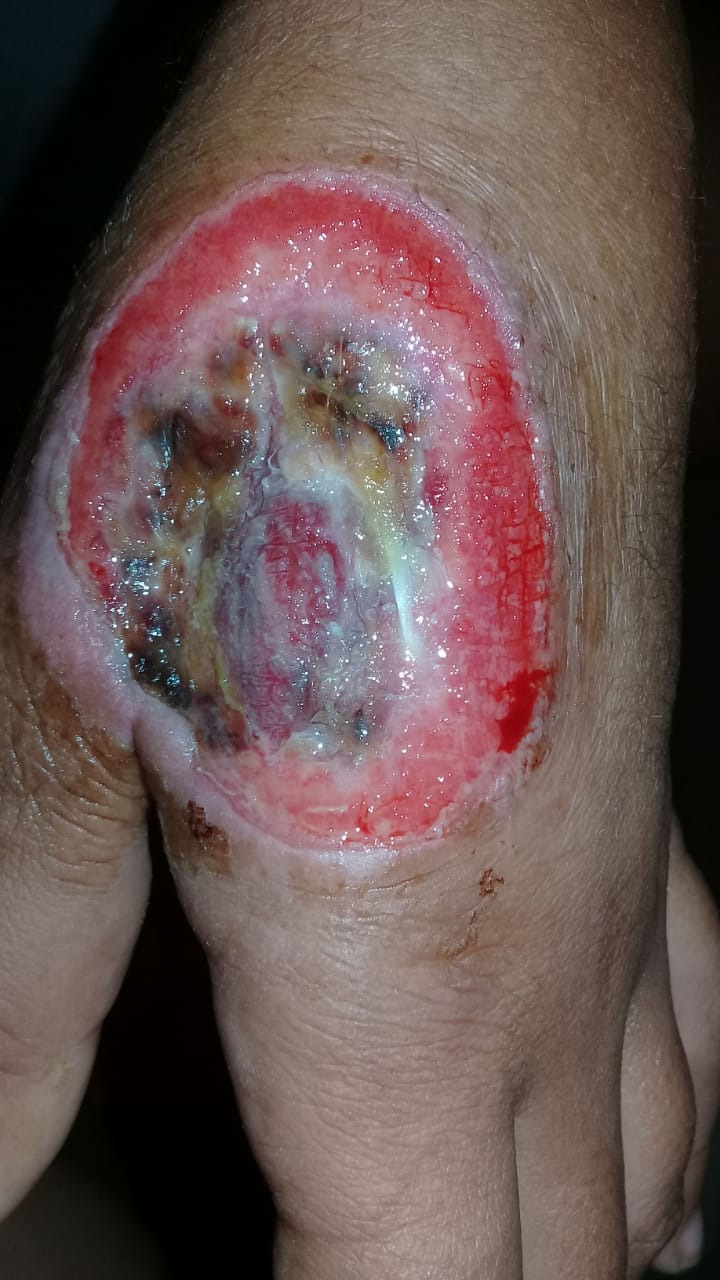
Postoperative image, showing the bleeding wound with signs of granulation.

At our service, we performed debridement of the wound, which bled and showed initial signs of granulation. There was relief from pain and the patient was discharged from hospital (Figure 1).

**Figure 2:**
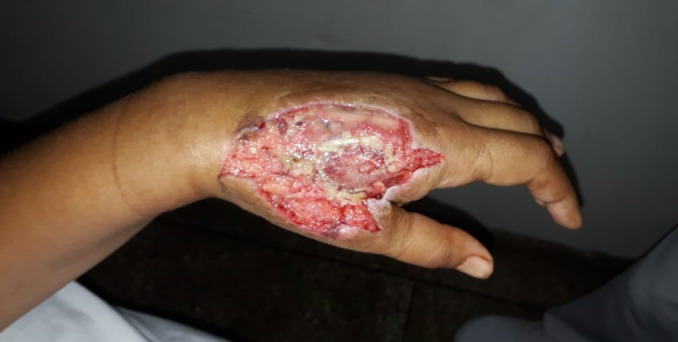
Exposure of the dorsal interosseous muscles, with points of necrosis.

Three days later, she returned complaining that the pain had worsened once more and necrotic crust covering the wound was observed again. She was admitted for immediate surgical intervention under brachial plexus anesthesia. Deep exploration of the wound, with dissection of the dorsal fascia to expose the dorsal interosseous muscles revealed that they were pale with points of necrosis that herniated as dissection of the fascia progressed, making it necessary to resect the necrotic musculature. The wound was extended in all directions to explore other muscles, seeking signs of damage (Figure 2).

**Figure 3:**
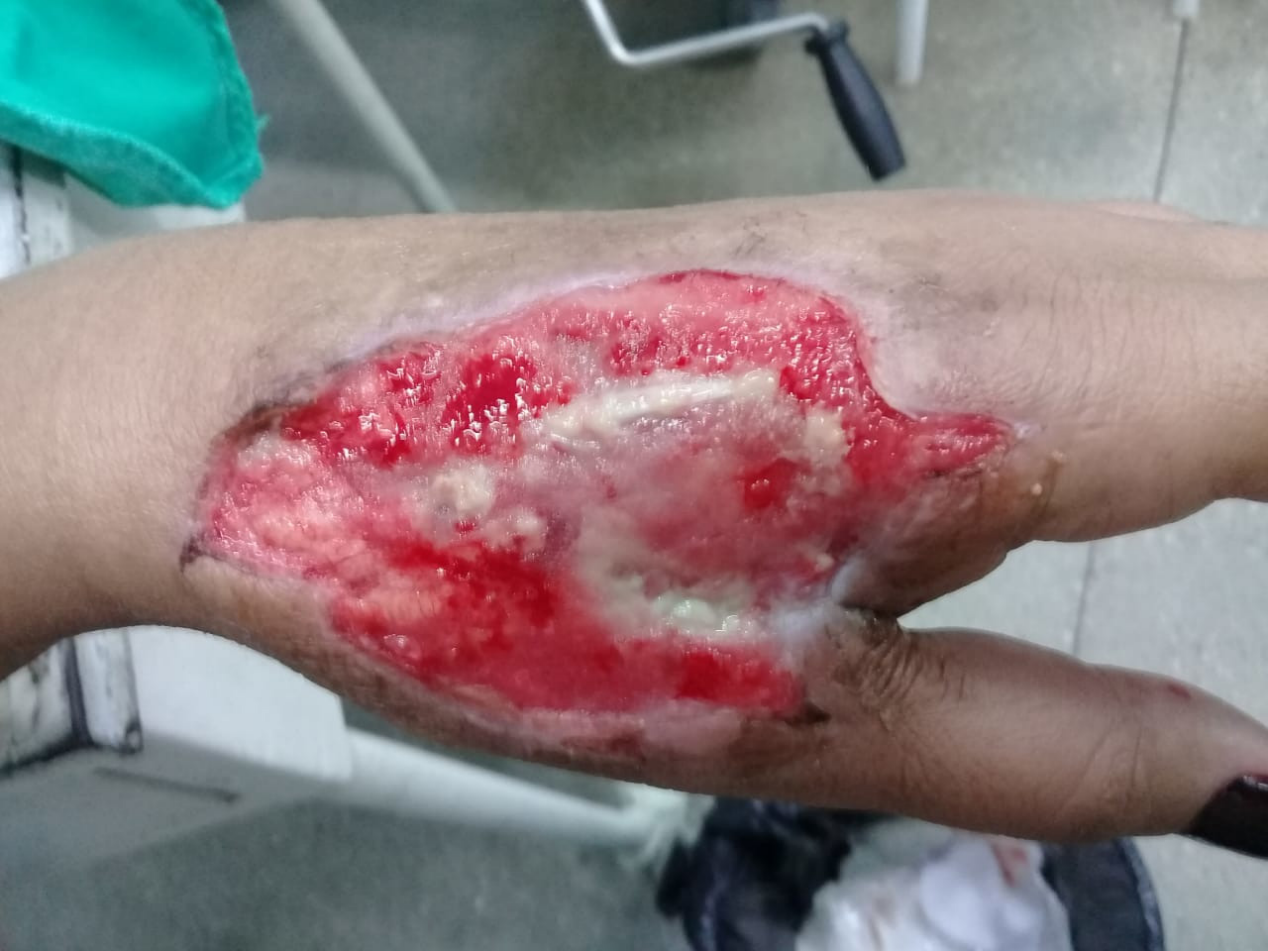
Patient reported improvement of pain and analgesic medication was withdrawn.

**Figure 4:**
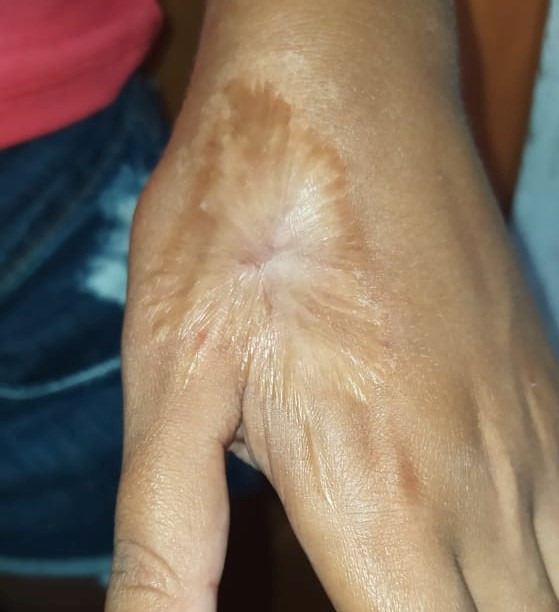
Healed wound in the region anterior of the anatomical snuffbox, with fine scar tissue and wrinkling at the edges, but the wound is fully healed over.

The patient’s postoperative course was pain free and she did not need analgesic medications. There were signs of granulation of the wound. She was discharged from hospital after 48 hours. One week later she returned to the service and was referred for motor physiotherapy and for a plastic surgery procedure to perform a split-thickness skin graft (Figure 3). Figure 4 shows the healed wound after the surgical procedures.

## DISCUSSION

The pathophysiological mechanism underlying compartment syndrome in second-degree deep burns of third degree burns involving extremities such as hands and feet is revealed by increased tissue pressure in muscle compartments in conjunction with the classic signs of inflammation, redness and swelling.^1^ The resulting pressure increase compresses vascular and nerve structures, provoking signs (restricted movement, cyanosis, and hypoperfusion) and symptoms (intense pain and paresthesia) compatible with the condition of reduced blood supply to the body part and immobilization. Disproportional pain that is resistant to conventional analgesic treatment in conjunction with pronounced edema are the foundation of establishing a clinical diagnosis of compartment syndrome, despite being generic symptoms that are present in countless trauma contexts.^2^

One study showed that achieving 90% release of the fascia can trigger the desired effect of sufficiently relieving intracompartmental pressure to reduce it to a level close to baseline.^3^ Thus, the vascular surgeon’s objective should be to decompress the muscle compartment involved using fasciotomy, thereby reestablishing functional circulation, since after a certain period this procedure will often be ineffective for achieving significant clinical improvement, with functional or anatomic loss of the limb affected by the syndrome.^4^ Therefore, early diagnosis of compartment syndrome or other potentially aggravating situations in patients who have been the victims of burns is clearly extremely important, as is implementation of measures to alleviate the patient’s condition, such as debridement of necrotic tissues, drainage of abscesses, and fasciotomy.

This syndrome has an incidence of 3.1 in every 100,000 inhabitants. It can be caused by increased volume inside a compartment (due to fractures, vascular injury, or damage to soft tissues), or by reduction in the volume of the compartment (burns and hernia repair at points of fragility).^5^ In the case described here, the burn victim had a reduced compartment volume and is therefore included in the annual incidence of acute compartment syndrome in traumatized extremities (7.3 per 100,000 men and 7.0 per 100,000 women).^6^ In terms of the number of complications such as compartment syndrome, amputations, rhabdomyolysis, and acute renal failure, these are common among electrical trauma victims, with rates of 72.3% for use of debridement, and 58.2% for skin grafts. The mean age of the victims, at 30.4 years, is similar to that of the patient in the case described (29 years), while the mortality rate is 8.2%.^7^

Consequently, when faced with a patient with a similar array of symptoms to those described here, the first step is to recognize the syndrome, make an early diagnosis and manage the disease in a practical and effective manner. In this context, in terms of the most appropriate treatment, decompressive fasciotomy offers good results, since it acts on the syndrome’s principal trigger factor, i.e., the increased intracompartmental pressure.^2^ It is also essential to take all precautionary measures, such as implementation of appropriate antibiotic therapy and exploration of the entire compartmental region to check for presence of the syndrome in the entire injured segment.^7^ The measures needed should be understood by the entire multi-professional healthcare team, since pathological progression of this clinical presentation can lead to more serious outcomes, up to and including death.

This article should serve as a warning to all physicians, and especially to surgeons, since they deal with this pathology constantly, highlighting the importance of recognition of the presence of the syndrome in areas that are not normally involved. Medical professionals should therefore be alert to the signs presented by the patient and aware of the correct and effective approach to management of the syndrome, targeting resolution of the pathological condition.

## CONCLUSIONS

This article illustrates management of a 29-year-old female patient who was initially admitted with high intensity pain in the left hand after suffering a hot coffee scald. Physical examination revealed a large injury with a rough crust at the level of the area anterior of the anatomical snuffbox and functional weakness of the fingers of the hand involved. Since this was a severe case of compartment syndrome, the wound was debrided, but the patient returned after her pain worsened. Deep wound exploration was initiated immediately and the dorsal fascia of the hand was opened. The results confirm the efficacy of surgical repair.
