# Supplementary material for: Compartment syndrome in the first dorsal interosseous compartment: case report
Source: J Vasc Bras. 2021 Apr 2;20:e20200094. doi: 10.1590/1677-5449.200094 (PMC8147704; doi:10.1590/1677-5449.200094)
Supplement: Translation

# Translation: Síndrome compartimental no primeiro interósseo dorsal: relato de caso

**Authors:** Clara Wilma Fernandes Rosendo, José Rodolfo Lopes de Paiva Cavalcanti, Raimundo Rosendo de Oliveira

**Affiliations:** 1 Universidade Federal do Rio Grande do Norte – UFRN, Departamento de Ciências da Saúde, Natal, RN, Brasil.; 2 Universidade do Estado do Rio Grande do Norte – UERN, Mossoró, RN, Brasil.; 3 Hospital Regional Tarcísio Maia – HRTM, Departamento de Cirurgia Geral e Vascular, Mossoró, RN, Brasil.

**Keywords:** síndromes de compartimento, fasciotomia, queimaduras

## Abstract

O diagnóstico da síndrome compartimental em regiões que não são frequentemente afetadas, por possuírem uma boa relação continente-conteúdo, pode se tornar difícil, uma vez que o cirurgião terá dificuldades em alcançar um diagnóstico apenas por um sinal ou sintoma isolado. Assim, muitas vezes, pode-se protelar a conduta adequada, desencadeando danos ao paciente. A paciente era uma mulher, de 29 anos, que foi atendida com dor em mão esquerda por queimadura, com lesão em região anterior à tabaqueira anatômica de tamanho significativo. A paciente já havia sido submetida a cirurgia na sua cidade de origem com desbridamento de pele e tecido subcutâneo há 30 dias, com evolução sem melhora do quadro doloroso. Estava em uso de antibioticoterapia (ceftriaxona 1 g de 12 em 12 horas) e analgesia com dose terapêutica de morfina de 8 em 8 horas.

## INTRODUÇÃO

As referências acerca da síndrome do compartimento tratam do envolvimento de estruturas musculares e nervosas fragilizadas por disfunções motoras e sensoriais. Esse quadro patológico refere-se a uma desproporção entre continente e conteúdo, devido à qual essas estruturas sofrem injúria, levando a sequelas irreversíveis de acordo com o grau de comprometimento caso não sejam tratadas adequadamente e em tempo hábil.

Os cirurgiões vasculares estão acostumados a vivenciar essa patologia principalmente em membros superiores e inferiores. Porém, qualquer região do corpo é passível de ser acometida por essa síndrome. O diagnóstico resulta de uma avaliação clínica cuidadosa e normalmente dispensa métodos diagnósticos especializados, com o intuito predominante de evitar graves complicações, a exemplo da inviabilidade motora.

## DESCRIÇÃO DO CASO

Esse estudo atendeu a todas as recomendações preconizadas pela resolução 466/2012 - CNE. Foi devidamente avaliado e aprovado pelo Comitê de Ética em Pesquisa (CAAE 31017920.7.0000.5294 e protocolo 4.050.507).

Uma paciente do sexo feminino, de 29 anos, deu entrada em um pronto-socorro queixando-se de dor intensa na mão esquerda por queimadura com café quente. No momento em uso de morfina para alívio do sintoma, apresentou ao exame físico lesão ao nível da região anterior à tabaqueira anatômica de tamanho significativo com crosta áspera cobrindo toda a lesão, associada a sinais de edema na mão e impotência funcional dos quirodáctilos, além de reação de dor de forte intensidade à manipulação.

Há quatro semanas, a paciente já havia sido submetida à desbridamento cirúrgico na sua cidade de origem, porém não apresentou melhora do quadro doloroso e estava em uso contínuo de morfina, sem resultados satisfatórios. Não havia sinais de infecção local.

Em nosso serviço, realizamos novo desbridamento da lesão, a qual apresentou-se sangrante, com sinais iniciais de granulação e alívio das dores, recebendo alta hospitalar em seguida (Figura 1).

**Figura 1:** Imagem pós-operatória mostrando lesão sangrante e com sinais de granulação.

Após três dias, houve retorno ao consultório por agravamento da dor e foi observada nova crosta necrótica cobrindo a ferida. Procedeu-se com internamento da paciente, para imediata conduta cirúrgica sob anestesia de plexo braquial, exploração profunda da ferida e abertura da fáscia dorsal da mão para exposição dos músculos interósseos dorsais, nos quais foi observado aspecto pálido e pontos de necrose que herniavam ao progredir com a abertura da fáscia, havendo necessidade de ressecção da musculatura necrótica. A lesão foi ampliada em todos os sentidos para exploração de outros músculos em busca de algum comprometimento (Figura 2).

**Figura 2:** Exposição dos músculos interósseos dorsais, com pontos de necrose.

No pós-operatório da paciente, houve evolução sem dores, sem uso de medicamentos analgésicos e com apresentação de sinais de granulação na ferida. Ela recebeu alta hospitalar depois de 48 horas. Após uma semana, a paciente retornou para o consultório, sendo encaminhada para fisioterapia motora e para procedimento de cirurgia plástica para preenchimento de enxerto dermoepidérmico (Figura 3). A Figura 4 mostra a lesão cicatrizada após os procedimentos cirúrgicos.

**Figura 3:** Paciente relatou melhora dos sintomas álgicos, e a medicação analgésica foi suspensa.

**Figura 4:** Ferida cicatrizada na região anterior à tabaqueira anatômica com tecido cicatricial fino mostrando enrugamento dos bordos, porém totalmente cicatrizada.

## DISCUSSÃO

O mecanismo fisiopatológico da síndrome do compartimento em queimaduras de segundo grau profundas ou de terceiro grau envolvendo extremidades, como mãos e pés, pontua um aumento da pressão tissular nos compartimentos musculares associado aos sinais clássicos de inflamação de rubor e edema^1^. Como resultado, a elevação pressórica observada comprime estruturas vasculares e nervosas, gerando sinais – restrição ao movimento, cianose e hipoperfusão – e sintomas – dor intensa e parestesia – condizentes com o quadro resultante de redução da irrigação sanguínea do membro e imobilidade. Um quadro álgico desproporcional e resistente ao tratamento analgésico convencional associado ao edema pronunciado constitui a base para o estabelecimento do diagnóstico clínico da síndrome compartimental, apesar de serem sintomas genéricos presentes em inúmeros contextos de trauma^2^.

Um estudo apontou que a obtenção de 90% de liberação fascial pode acarretar um efeito desejado na diminuição da pressão intracompartimental a ponto de atingir um valor próximo ao basal^3^. Assim, mediante a descompressão do compartimento muscular comprometido via fasciotomia, o propósito do cirurgião vascular deve ser restabelecer a circulação funcional, visto que, depois de determinado período, a realização desse procedimento frequentemente torna-se ineficaz para a obtenção de uma melhora clínica considerável, podendo surgir como sequela a perda funcional ou anatômica do membro afetado pela síndrome^4^. Logo, pontua-se de extrema importância o diagnóstico precoce da síndrome compartimental em um paciente vítima de queimadura e outras situações possivelmente agravantes, além da implementação de medidas para o alívio da situação do paciente, como o desbridamento de tecidos necrosados, a drenagem de abscessos e a fasciotomia.

Essa síndrome apresenta uma incidência de 3,1 a cada 100.000 habitantes. Entre as suas causas, caracteriza-se o aumento do volume dentro de um compartimento (causado por fraturas, lesão vascular e de tecidos moles), bem como a redução desse volume (queimaduras e reparação de hérnias em pontos frágeis)^5^. No caso relatado, a paciente vítima de queimadura apresentava redução do volume compartimental, estando incluída na incidência anual dessa síndrome aguda em extremidade traumatizada (7,3 por 100.000 homens e de 7,0 por 100.000 mulheres)^6^. Relativamente ao número de complicações como síndrome compartimental, amputações, rabdomiólise e insuficiência renal aguda, a existência dessas foi frequente entre as vítimas de trauma elétrico, com uma taxa de 72,3% de técnicas de desbridamento e 58,2% de enxertos de pele realizados. Acrescenta-se que, semelhante à paciente do caso relatado (29 anos), a idade média das vítimas foi de 30,4 anos, e a taxa de mortalidade foi de 8,2%^7^.

Por conseguinte, diante de um paciente com contexto sintomatológico semelhante ao estudado, o primeiro passo consiste em reconhecer a síndrome, realizar o diagnóstico precocemente e manejar a doença de forma prática e resolutiva. A partir disso, como tratamento mais apropriado, a fasciotomia descompressiva apresenta bons resultados, pois atua contra o principal fator desencadeante da síndrome, ou seja, o aumento da pressão compartimental^2^. Também é essencial aplicar todas as condutas de precaução, a exemplo da implementação de antibioticoterapia adequada e exploração de toda a região compartimental para verificação da presença da síndrome em todo o segmento lesionado^7^. As medidas a serem tomadas devem ser compreendidas por toda a equipe multiprofissional de saúde, visto que a evolução patológica desse quadro clínico pode levar a desfechos mais graves e até à morte.

O presente trabalho serve de alerta a todos os médicos – especialmente cirurgiões, por lidarem constantemente com essa patologia – para a importância de reconhecer a presença da síndrome em regiões não comumente acometidas. Desse modo, o profissional médico deve estar atento aos sinais apresentados pelo paciente, bem como ao manejo correto e eficaz dessa doença, visando à resolução desse quadro patológico.

## CONCLUSÃO

O presente trabalho ilustra a conduta referente a uma paciente feminina, de 29 anos, inicialmente admitida com quadro álgico de forte intensidade em mão esquerda após queimadura com café quente. O exame físico demonstrou lesão ao nível da região anterior à tabaqueira anatômica de tamanho significativo com crosta áspera e fragilidade funcional dos dedos da mão acometida. Por se tratar de um quadro grave de síndrome compartimental, foi realizado o desbridamento da região, porém houve retorno da paciente com piora da dor, sendo realizada imediata exploração profunda da ferida e abertura da fáscia dorsal da mão, corroborando a eficiência do reparo operatório.
